# Exploring the relationships among writing enjoyment, cognitive load, and academic achievement in L2 project-based writing

**DOI:** 10.3389/fpsyg.2026.1882516

**Published:** 2026-09-23

**Authors:** Guohua Li, Chunyan Wang

**Affiliations:** School of Languages, Shanghai University of International Business and Economics, Shanghai, China

**Keywords:** cognitive load, integrated model of cognitive-affective learning (IMCAL), project-based writing, writing achievement, writing enjoyment

## Abstract

Although project-based writing (PBW) is widely implemented in second language (L2) teaching context, its high cognitive demands are frequently reported. Drawing on the Integrated Model of Cognitive-Affective Learning (IMCAL), this study investigated how writing enjoyment interacted with cognitive load in shaping L2 learners’ PBW achievement. This study employed a mixed-methods design, incorporating survey data from 417 Chinese university students and follow-up semi-structured interviews. Structural equation modeling (SEM) was used to test a mediation model in which cognitive load mediates the relationship between writing enjoyment and writing achievement. Results indicated that writing enjoyment not only directly and positively predicted writing achievement, but also attenuated the negative relationship between cognitive load and writing achievement. Qualitative results further explained the mechanisms through which writing enjoyment was associated with cognitive load and writing achievement. Critical antecedents of writing enjoyment in PBW learning environments were also explored. These results yield practical implications for PBW course design, as well as theoretical insights into understanding IMCAL in the L2 PBW context.

## 1 Introduction

In recent years, educational researchers have shifted their attention from the impact of negative emotions to positive emotions on second language learning (Dewaele and MacIntyre, 2014; Tahmouresi and Papi, 2021). Positive emotions, including joy, interest, contentment, pride, and love, broaden individuals’ momentary thought-action repertoires, build enduring personal resources (Fredrickson, 1998, 2003), facilitate problem-solving, and shape goal-directed behavior (Pekrun et al., 2002). Despite their significance, learners’ positive emotional experiences remain underexplored in complex learning environments characterized by high cognitive demands, such as project-based writing (PBW). Although widely adopted in higher education, particularly in Asia, PBW tasks are frequently reported to pose substantial challenges for learners (Wang et al., 2023; Wang et al., 2024a).

Cognitive load (CL) refers to the mental effort required to process information during learning (Sweller, 1988). Traditionally, cognitive load has been conceptualized primarily in terms of task-related demands; however, recent research suggests that it is also shaped by learner-related factors, including emotional experiences (Schrader and Kalyuga, 2022). Over the past two decades, advances in neuroscience have provided increasing evidence for the close interconnection between cognition and emotion, demonstrating that affective and cognitive processes are deeply intertwined and dynamically interact during learning (Pessoa, 2008). This cognition- emotion connection is reflected in the Integrated Model of Cognitive-Affective Learning (IMCAL), which extends cognitive load theory by incorporating learners’ emotions into the learning process (Plass and Kaplan, 2016). Nevertheless, empirical research testing the applicability of the IMCAL framework in L2 writing remains limited, particularly in the cognitively demanding context of PBW.

To address this research gap, the present study draws on the IMCAL framework to examine the relationships among writing enjoyment, cognitive load, and L2 writing achievement. Quantitative analyses were conducted to investigate the extent to which the IMCAL framework can account for these relationships in the L2 PBW context. Qualitative data were further incorporated to provide contextualized interpretations of the quantitative findings and to explore the antecedents of writing enjoyment. Through this mixed-methods approach, the study seeks to provide empirical insights into the role of writing enjoyment in relation to cognitive load during complex writing tasks. It also aims to offer pedagogical implications for the design of effective PBW instruction.

## 2 Literature review

### 2.1 Cognitive load and positive affect

Cognitive load (CL) is the mental effort required to process interacting information elements in working memory during learning (Sweller, 1994). It is commonly categorized into three types: intrinsic, extraneous, and germane load (Plass and Kalyuga, 2019; Sweller et al., 1998). Intrinsic load refers to the cognitive demands imposed by the inherent complexity of the material (Sweller, 1988). Extraneous load arises from task-irrelevant processes that do not contribute to learning (Kalyuga, 2011; Sweller, 1988). Germane load reflects the cognitive resources devoted to schema construction (Sweller et al., 1998).

Within the traditional framework of cognitive load theory (CLT), emotion has received limited attention as a component of information processing. However, advances in affective neuroscience have demonstrated the close interdependence of emotion and cognition, suggesting that both arise from a common cortical system (LeDoux and Brown, 2017). Building on this perspective, Plass and Kaplan (2016) proposed emotion as a distinct processing channel within CLT in their Integrated Model of Cognitive-Affective Learning (IMCAL). Within the IMCAL framework, positive affect is proposed to interact with cognitive load through several key mechanisms. First, it may reduce extraneous load by promoting more efficient cognitive processing and minimizing distracting thoughts, such as self-doubt or fear of failure, thereby freeing working memory resources (Plass and Kalyuga, 2019). Second, positive affect may enhance core cognitive processes, including attention, encoding, and retrieval, often through neurocognitive mechanisms that support flexible thinking and memory consolidation, which enhances effective learning and reduces the perception of intrinsic cognitive load (Plass and Kalyuga, 2019). Third, it may modulate perceptions of intrinsic load by making complex tasks feel more manageable. Although it does not alter objective task complexity, positive affect supports emotional regulation and persistence, enabling learners to cope more effectively with high intrinsic demands (Plass and Kalyuga, 2019). Finally, positive affect may enhance intrinsic motivation and cognitive engagement, thereby increasing learners’ investment of mental effort (Plass and Kalyuga, 2019). Through this increased engagement, learners may construct new schemas, which can reduce the intrinsic cognitive load associated with task element interactivity (Kalyuga, 2011).

Empirical research on the interaction between positive affect and cognitive load remains limited. In reading contexts, Cai et al. (2024) found that enjoyment negatively predicted both intrinsic and extraneous cognitive load, thereby indirectly enhancing achievement. In L2 writing, Tabari et al. (2024) reported that higher levels of writing enjoyment among primary students were associated with lower cognitive load and better use of working memory for linguistic development. Similarly, Xu et al. (2020) demonstrated that growth mindset, a form of positive affective disposition (Yeager and Dweck, 2020), indirectly influenced academic performance by reducing intrinsic and extraneous cognitive load. However, to date, no study has systematically examined whether positive affect can influence cognitive load in the PBW context which is notoriously challenging.

### 2.2 Writing enjoyment, antecedents and writing achievement

Writing enjoyment refers to the positive emotion of pleasure and enjoyment experienced during writing activities (Zumbrunn et al., 2019). Writing enjoyment plays a particularly important role in cognitively demanding tasks which requires sustained effort, strategic thinking, and high levels of self-regulation (Graham and Harris, 2000; Zimmerman and Riesemberg, 1997). Hayes (1996) pioneered the view that writers rely on both cognitive and affective resources to engage in the cognitive and goal-oriented process of writing. L2 writers with stronger positive emotions are more likely to be prepared to meet the cognitive demands involved in writing, such as careful planning and frequent monitoring, and may achieve higher writing performance (Li et al., 2023).

Research on the antecedents of enjoyment in PBW remains limited, despite its importance. Grounded in Pekrun’s (2006) control-value theory of achievement emotions, academic enjoyment is posited to emerge when learners make positive control and value appraisals of an activity. Learner traits (e.g., cognitive abilities) and situational factors (e.g., task autonomy, positive feedback) can also indirectly shape achievement emotions. In second language learning, situational antecedents such as a positive classroom atmosphere, teacher support, constructive feedback, and autonomous tasks have been found to foster foreign language enjoyment (Botes et al., 2021; Dewaele and MacIntyre, 2014). In technology-supported academic writing, learners’ acceptance of AI tools may represent an additional antecedent of writing enjoyment. Learners’ perceptions and adoption of AI technologies may contribute to a deep approach to learning (Sun et al., 2025) and enjoyment in technology-mediated writing (Sun et al., 2026b). Notably, academic emotions are domain-specific (Putwain et al., 2018); therefore, their antecedents should be examined within the learning context rather than generalized across learning environments.

Empirical findings on the relationship between writing enjoyment and writing achievement are generally positive, but remain inconsistent. A series of studies by Graham and colleagues with elementary students (Graham et al., 2012; Graham et al., 2017) consistently found that higher levels of writing enjoyment were associated with longer and higher-quality texts. However, contradictory evidence also exists; for instance, Olinghouse and Graham (2009) reported a nonsignificant relationship between writing enjoyment and writing proficiency.

### 2.3 Cognitive load, writing achievement and PBW tasks

Cognitive load theory posits that working memory has a limited processing capacity; consequently, learning becomes less effective when cognitive demands exceed this capacity (Sweller, 1988, 1994). Under conditions of excessive cognitive load, cognitive resources are diverted away from schema construction and task-relevant processing, thereby reducing learning efficiency (Plass and Kalyuga, 2019). Accordingly, higher levels of cognitive load are generally associated with less favorable learning outcomes, particularly in tasks that impose substantial cognitive demands.

L2 writing is one such cognitively demanding activity because it requires learners to coordinate multiple processes simultaneously, including idea generation, linguistic encoding, text organization, and revision, while monitoring language accuracy and rhetorical appropriateness (Kellogg, 2008). The concurrent management of these processes places considerable demands on working memory, making L2 writing especially susceptible to cognitive overload.

A growing body of empirical research supports the association between cognitive load and L2 writing performance. Using psychophysiological measures, Lee et al. (2022) reported that higher cognitive load was associated with poorer online L2 writing performance. Jiang and Kalyuga (2022) further found that collaborative writing was associated with lower cognitive load and better writing quality, suggesting that reducing unnecessary cognitive demands may facilitate L2 writing. Similarly, Wang et al. (2024b) reported that cognitive load was associated with writing performance both directly and indirectly through writing anxiety. Collectively, these studies suggest that cognitive load constitutes an important psychological factor related to L2 writing achievement.

Over the past decade PBW has been increasingly implemented in Asian educational contexts, although substantial challenges met by L2 learners have been frequently reported (Wang et al., 2023; Wang et al., 2024a). As a learner-centered approach, PBW positions students as active agents who independently identify, explore, and resolve problems. The PBW process typically involves formulating research questions, reviewing academic literature, designing research methods, collecting and analyzing data, and composing formal written reports. Compared with traditional language instruction, its strong emphasis on learner autonomy, together with limited instructional time, imposes considerable cognitive demands on students and may lead to cognitive overload. Therefore, alleviating cognitive load in PBW tasks is critical for effective pedagogy. Although emerging evidence suggests that positive emotions can reduce cognitive load, their regulatory function remains underexplored in PBW research and instructional practice.

The above literature review reveals several gaps that need to be addressed. First, while theoretical frameworks have acknowledged the interplay between positive affect and cognitive load, empirical investigations into how writing enjoyment interacts with different types of cognitive load to shape L2 writing achievement are scarce, especially within the cognitively demanding PBW context. Second, although the antecedents of language learning enjoyment have been discussed in general classroom settings, context-specific factors contributing to writing enjoyment in PBW remain underexamined, leaving limited practical guidelines for instructional design. Accordingly, the following research questions are proposed:

1. To what extent does writing enjoyment predict cognitive load in PBW activities?
2. To what extent does writing enjoyment predict PBW achievement directly and indirectly through cognitive load?
3. What are the key antecedents of writing enjoyment in PBW tasks?

## 3 Methods

Following other L2 writing studies which combine structural equation modelling and interviews to unpack the psychological mechanisms underlying quantitative pathways (e.g., Sun et al., 2026a), the study adopted similar approach, integrating quantitative questionnaire data with qualitative insights from semi-structured interviews.

### 3.1 Participants

The quantitative phase of the study involved a sample of 417 undergraduate students enrolled in academic writing courses at a Chinese university specializing in economics and business studies. The course was delivered using the PBW approach, requiring students to independently select research topics and complete an academic report over the course of a semester, with instructors providing structured scaffolding. Participants’ English proficiency ranged from high B1 to high C1 according to the Common European Framework of Reference for Languages (CEFR). The sample had a mean age of 20.5 years (range = 19–22) and comprised 162 male and 255 female students, a gender distribution reflecting the institution’s overall demographic profile, which features a higher proportion of female students. Participation in the survey was voluntary.

To complement the quantitative findings with qualitative insights, eight students were recruited from the PBW course to participate in interview. Specifically, four students with the highest writing performance and four with the average writing performance were intentionally recruited to represent variation in learning experiences and perceptions. An interview sample of eight participants was considered appropriate because previous research has shown that thematic saturation can often be achieved within approximately 6–12 interviews in relatively homogeneous samples (Guest et al., 2006). Demographic profiles of the interviewed students are presented in Table 1.

**Table 1:** Demographic profiles of the interviewed students.

Name	Major	Gender
JM	Law	Female
CS	Law	Male
LJ	International organization	Male
YW	Economics	Female
XC	Economics	Female
YF	Economics	Female
JC	Business Law	Female
YL	Business Law	Male

### 3.2 Data collection procedures

The questionnaire data were collected from the participants in July 2024. The course spanned from early February to early July, during which students conducted small-scale research in groups of three to four and submitted individual academic reports of approximately 3,000 words at the end of the semester. Upon completion of the course, the questionnaire was administered via an online survey platform (Wen Juan Xing, Changsha Ranxing Company). Participants were explicitly informed of the study’s purpose and assured that participation was voluntary. The confidentiality and anonymity of their responses were ensured. Participants provided informed consent prior to completing the questionnaire.

In the subsequent qualitative phase, semi-structured interviews were conducted to serve a dual purpose: first, to explain the quantitative relationships among writing enjoyment, cognitive load, and writing achievement; and second, to explore the antecedents of writing enjoyment in the L2 project-based writing context. The interviews were conducted in Chinese and lasted 40–45 min each, ensuring the collection of sufficiently detailed and credible data.

### 3.3 Measurement instruments

#### 3.3.1 Writing enjoyment

Students’ enjoyment of project-based writing was assessed with an adapted version of the Foreign Language Enjoyment (FLE) scale originally developed by Dewaele and MacIntyre (2014). The original items were modified to align with the PBW context. Participants indicated their level of agreement with three statements on a 6-point Likert scale (1 = strongly disagree, 6 = strongly agree). The three items were: ‘I like project-based writing’ (*M* = 3.98, SD = 1.20), ‘I like attending writing classes’ (*M* = 3.94, SD = 1.15), and ‘I like to devote my time to project-based writing’ (*M* = 3.76, SD = 1.20). The internal consistency of this three-item scale was satisfactory (Cronbach’s α = 0.86). The standardized factor loadings for the three items ranged from 0.80 to 0.84, exceeding the recommended threshold of 0.50 (Hair et al., 2021), providing support for the reliability of the measurement.

#### 3.3.2 Cognitive load in academic writing tasks

The cognitive load associated with the research report writing task was measured using a 13-item subjective self-report instrument adapted from the Project-Based Academic Report Writing Challenges Scale developed by Wang et al. (2023). Participants rated the perceived difficulty of various aspects of the writing process on a 6-point Likert scale (1 = not difficult at all, 6 = very difficult). The 13 items can be categorized into two subscales: (a) idea-planning load, comprising 6 items assessing the cognitive load during information seeking, academic reading/knowledge acquisition, and writing planning; and (b) writing load, comprising 7 items assessing the cognitive load during the translation of ideas into text and subsequent revision. The CFA supported the hypothesized two-factor measurement structure, which demonstrated good model fit (χ^2^/df = 2.411, RMSEA = 0.057, TLI = 0.961, CFI = 0.973, and SRMR = 0.040). The scale demonstrated good internal consistency (Cronbach’s α = 0.855 for idea-planning load, 0.887 for writing load, and 0.921 for the overall scale). For the subsequent SEM analysis, cognitive load was represented by an overall observed composite score. The mean score for each subscale was calculated, and the two subscale means were then averaged to obtain the overall cognitive-load score. The overall cognitive-load score was *M* = 4.00 (SD = 0.76). We chose to represent CL as an observed composite, rather than a latent variable, because our aim was to examine the relationship between WE and overall CL rather than to estimate separate structural relations for its two dimensions. This approach also allowed us to maintain a parsimonious SEM specification.

#### 3.3.3 Academic writing achievement

Following the self-assessment framework proposed by Pike (1996, 1999), participants were asked to evaluate their project-based writing (PBW) achievement using a single-item six-point Likert scale (1 = very low, 6 = very high). Self-reported writing achievement was used instead of course grades for two reasons. First, PBW reports were assessed by course instructors, each of whom evaluated their own students without inter-rater reliability checks. Second, self-reported academic performance has been widely recognized as a valid indicator of learning outcomes in educational research (Pike, 1996, 1999; Ross, 1998; Li et al., 2023). Pike (1996, 1999) showed that students’ self-reports demonstrate acceptable convergent validity with external measures of academic achievement. In second language research, Ross (1998) reported positive associations between learners’ self-assessments and external language proficiency measures. In our study, the correlation between self-reported writing achievement and the course-assigned writing score was *r* = 0.67, *p* < 0.05, suggesting acceptable convergent validity. Moreover, because the questionnaire was administered anonymously (i.e., without collecting names or student IDs), social desirability bias was reduced, thereby increasing the credibility of the self-reported writing achievement (Pike, 1996, 1999). The mean self-reported writing achievement in the present sample was 4.02 (SD = 1.07).

### 3.4 Semi-structured interviews

The semi-structured interview was organized around three sections. The first section sought to investigate whether and how writing enjoyment influenced participants’ cognitive load. A representative prompt was: “During the PBW task, did your interest in the topic make the task feel less demanding?” The second part explored whether positive emotions increased learners’ investment of effort or use of strategies, thereby potentially influencing writing achievement. A sample question was: “What motivates you to devote additional time and energy to your project research: interest, a sense of accomplishment, responsibility to your team, or other factors?” The third section aimed to identify the key antecedents of writing enjoyment within the PBW context. For example, participants were asked: “What factors in course design, instructor guidance, or group collaboration made the PBW task enjoyable for you?”

### 3.5 Statistical analysis

We took two steps to apply a structural equation modeling (SEM) analysis in the study. It is believed that SEM analysis can identify sources of the misfit of data models and prevent problems of nonconvergence (Mueller and Hancock, 2008). Firstly, we evaluated the measurement models of the questionnaire. The reliability and validity of the questionnaire were checked with CFAs using software—AMOS program Version 22.0 (Arbuckle, 2013). This was designed to check whether the data we collected fit the hypothesized measurement models. Informed by the CFA results, we proposed a mediation model and evaluated it by SEM. Several goodness of fit indices was reported in order to check model fits, including the ratio of chi-square (χ^2^) divided by the degree of freedom (df), the comparative fit index (CFI), the Tucker-Lewis index (TLI), the root mean square error of approximation (RMSEA), and the standardized root mean square residual (SRMR). We applied Hu and Bentler’s (1999) cut-off values and found that the CFI and TLI were lower than 0.95; RMSEA lower than 0.06; and SRMR no larger than 0.08, showing a good model fit. In addition, chi-square/df ratio values lower than 2.5 were considered in our study to represent an acceptable model (Byrne, 1991).

Interview data were analyzed using thematic analysis following the phased procedure outlined by Braun and Clarke (2021). After the qualitative analysis was completed, the emergent themes were triangulated with the quantitative findings to synthesize the study’s major findings.

## 4 Results

### 4.1 Descriptive statistics

Table 2 presents the descriptive statistics and correlations for writing enjoyment, cognitive load, and writing achievement. Consistent with these results, writing enjoyment was negatively associated with cognitive load and positively associated with writing achievement. Cognitive load was negatively associated with writing achievement.

**Table 2:** Results of descriptive statistics and Pearson correlation analysis.

Variables	V1	V2	V3
V1.	1	−0.144^**^	−0.163^**^
V2.		1	0.242^**^
V3.			1
Mean	4.45	3.86	4.02
SD	1.04	1.05	1.07

V1, cognitive load; V2, writing enjoyment; V3, writing achievement; ^**^Correlation is significant at the 0.01 level (2-tailed).

### 4.2 Model evaluation

The next step was to specify a SEM to examine: (1) how writing enjoyment influenced cognitive load; and (2) how writing enjoyment influenced writing achievement, both directly and indirectly, being mediated by cognitive load. A critical condition for testing mediation effects is that significant relationships should exist between predictors and dependent variables (Hayes, 2013). Therefore, we examined the correlations among writing enjoyment, cognitive load, and academic writing achievement. Table 2 shows the correlations between the dependent and independent variables, providing sufficient justification for testing mediation effects among these constructs. Accordingly, a partial mediation model was proposed, including both direct and mediation paths from the predictors to the outcome variables (writing enjoyment → writing achievement; writing enjoyment → cognitive load → writing achievement). The structure of the hypothesized partial mediation model is shown in Figure 1.

**Figure 1:**
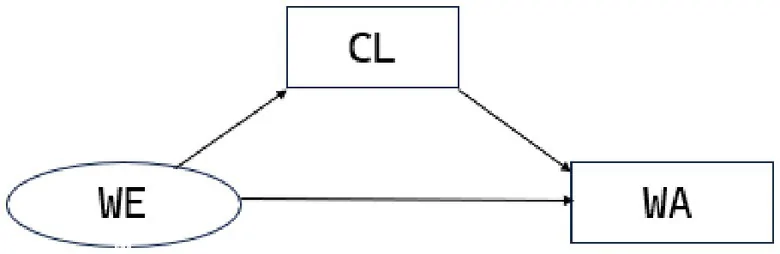
A proposed partial mediation model with both mediation and direct paths from writing enjoyment to writing achievement. WE, writing enjoyment (latent variable); CL, cognitive load (observed composite score); WA, writing achievement (observed score).

The SEM results indicated an acceptable model fit: χ^2^ (4) = 5.858, χ^2^/df = 1.46, *p* = 0.216 (> 0.05), CFI = 0.99, TLI = 0.98, RMSEA 90% confidence interval = 0.04 [0.00, 0.09], SRMR = 0.06. On this basis, we retained the partial mediation model of writing enjoyment on writing achievement via cognitive load. Figure 2 presents the standardized regression coefficients for the direct and indirect paths among writing enjoyment, cognitive load, and writing achievement. Overall, writing enjoyment exerted both a direct effect on writing achievement and an indirect effect through cognitive load. Specifically, the significant direct path was from writing enjoyment to writing achievement (*β* = 0.27, *p* < 0.01). Writing enjoyment also significantly predicted cognitive load (*β* = −0.17, *p* < 0.001), and cognitive load, in turn, significantly predicted writing achievement (*β* = −0.12, *p* < 0.01). In total, 14.70% of the variance in writing achievement was explained by the predictor variables.

**Figure 2:**
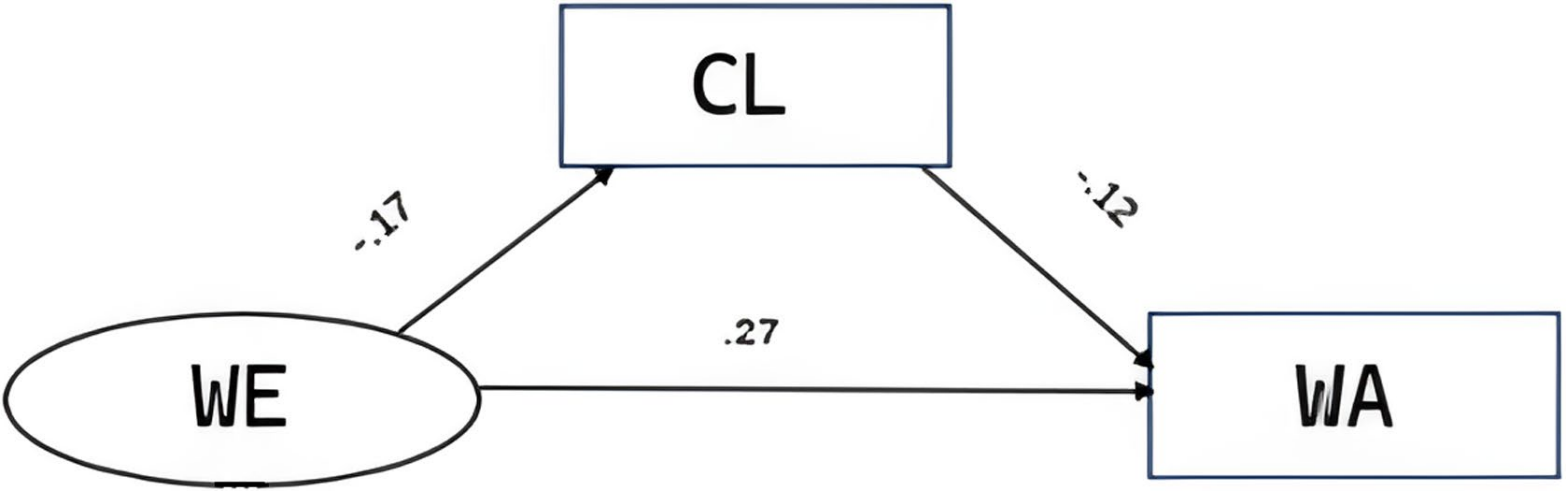
Results of the SEM. WE, writing enjoyment; WA, writing achievement; CL, cognitive load. All path coefficients are significant with *p* < 0.01.

To test the significance of the mediator effects, we used a bootstrapping procedure with bias-corrected confidence estimates. In this study, 95% confidence interval of the indirect effects was obtained using 2000 bootstrap resamples (Preacher and Hayes, 2008). The mediation path from writing enjoyment to cognitive load to writing achievement was significant (*β* = 0.019, *p* < 0.01), with a 95% confidence interval of [0.016, 0.057].

To summarize, writing enjoyment was negatively associated with cognitive load in this study, suggesting that writing enjoyment is likely to alleviate learners’ cognitive load. Second, writing enjoyment not only significantly and positively predicted writing achievement but also appeared to neutralize the negative association between cognitive load and writing achievement to some extent.

### 4.3 Qualitative analysis results

#### 4.3.1 Writing enjoyment and cognitive load

Interview data revealed that writing enjoyment alleviates learners’ perceived cognitive load by reducing extraneous cognitive load, enhancing cognitive processing, and modulating perceived intrinsic load.

First, participants consistently reported that enjoyment helped minimize task-irrelevant processing. Interest in the project acted as a positive driving force that helped them “stay focused and prevent … getting distracted by trivial matters” (YL). This sustained attention made them “less disturbed by distractions” throughout the writing process (JC). Furthermore, a determined and collaborative mindset for problem solving helped them overcome periods of anxiety that otherwise would have led to divided attention and lower efficiency (JM).

Second, positive affect was associated with more efficient and creative engagement with task-relevant processes. Students described how interest allowed them to “fully unleash [their] creativity” (CS), finding PBW “more enjoyable and liberating, with plenty of room for creativity” than conventional tasks (JM). Deep engagement, such as extensive reading on the topic, helped them “acquire more knowledge and clarify thinking” during complex stages like questionnaire design (YW), and was associated with inspired creative thinking (JM and YL).

Finally, positive affect modulated their perceived intrinsic cognitive load. Students noted that enjoyment in a sub-task (e.g., data analysis) made the overall project seem “a bit easier” and less tedious (LJ). When the topic was interesting, searching for materials felt “like a kind of entertainment” and thus less strenuous (JM). A positive mindset, confidence, and the belief that “nothing was insurmountable” made complex requirements easier to tackle (JM). The sense of accomplishment after overcoming challenges led to reflections such as “It wasn’t so difficult after all”, boosting future confidence (JC). Strategic approaches, such as breaking down complex problems into manageable steps, also reduced the perceived difficulty (YL).

#### 4.3.2 Writing enjoyment and writing achievement

Interview data revealed that enjoyment influenced achievement by enhancing motivation, engagement, and strategy use.

Interview data showed that enjoyment was a powerful source of motivation that drove deeper investment. Interest provided a “clear thread” that enabled smooth task completion (XC) and led students to “invest so much time and effort” (YF). Motivation stemmed from multiple sources: the intellectual value of exploring under-researched topics (YW), the perceived social relevance and impact of the work (YF and XC), positive team dynamics and a sense of shared responsibility (XC and YW), and the personal satisfaction of skill development and overcoming challenges (JM and LJ). For novice researchers, inherent interest and the desire to “do it well” were primary motivators (YL).

Writing enjoyment also promoted strategic engagement and persistence. This motivation translated into proactive and persistent learning behaviors. Students invested substantial time in all project phases out of a sense of responsibility (YF). They persevered in contacting research targets despite repeated rejections (LJ) and expended extra effort to secure sufficient survey respondents (JM). Because the process was “enjoyable and liberating,” they willingly invested additional time and energy without feeling internal resistance (JM). Team responsibility also helped maintain persistence during anxiety-provoking tasks like data analysis (YL).

Adaptive strategy use was also enhanced. Writing enjoyment was associated with the flexible and resourceful use of learning strategies. When standard approaches failed (e.g., contacting enterprises), students strategically sought alternative pathways (e.g., engaging government departments) (LJ). To understand complex issues, they engaged in extensive reading and group discussion to develop new analytical insights (JM). They actively sought resources to learn new skills, using online tutorials and AI tools to master software such as SPSS (YL). They also employed project management strategies like role division and deadline setting (JC) and consciously transferred writing skills learned in class to their project reports (YF).

#### 4.3.3 Identification of antecedents of writing enjoyment

The interview data revealed two interrelated clusters of factors associated with students’ writing enjoyment: learner factors and situational factors.

For learner factors, participants developed positive emotions during the learning process as a result of enhanced academic competence. Competence gains included mastery of analytical tools such as SPSS (YL), SPSSPRO and Stata (LJ), improved academic writing (YW), a sense of achievement from completing challenging research work (CS, JC, JM, YF and YL) and uncovering unexpected survey findings (CS and JM). Participants also stated that intrinsic curiosity regarding self-chosen topics served as an internal driver maintaining sustained writing engagement (JC, JM, YW, XC, YF, YL, CS and LJ). Such experiences demonstrate that writing enjoyment is reinforced when students perceive they are capable of overcoming obstacles and mastering new research-related skills.

Regarding situational factors, four recurring contextual factors emerged. First, participants valued greater autonomy in selecting personally relevant and socially meaningful research topics (JC, JM and LJ). Second, well-organized team collaboration with clear task divisions and timely feedback enhanced participants’ sense of confidence (JC, JM, YW, XC, YF, YL, CS and LJ). Third, participants reported greater enjoyment when they perceived their research as having practical value for industries or the wider public (YW, XC and LJ). Fourth, step-by-step instructor guidance and access to previous project samples helped reduce uncertainty throughout the writing process (JC, JM, YW, XC, YF and YL). These situational factors seemed to interact with learner factors in stimulating participants’ writing enjoyment.

## 5 Discussion

The following discussion synthesizes the SEM and interview findings to address the three research questions.

### 5.1 The association between writing enjoyment and cognitive load

In response to the first research question, the quantitative results showed that writing enjoyment was negatively associated with cognitive load (*β* = −0.17, *p* < 0.001), indicating that higher writing enjoyment was linked to lower cognitive load in both the idea planning and writing stages of PBW tasks. This finding is consistent with the Integrated Model of Cognitive-Affective Learning (Plass and Kaplan, 2016), which conceptualizes how positive affect interacts with cognitive load during learning.

The qualitative findings support this relationship by showing that students with higher writing enjoyment experienced fewer distractions and maintained stronger focus on core PBW activities (e.g., literature review, questionnaire design, and data analysis), suggesting reduced task-irrelevant processing and lower extraneous cognitive load. Second, writing enjoyment serves as a cognitive enhancer, supporting creative and deep thinking, which aligns with neuroscience perspectives on how positive affect can facilitate flexible cognition (Pessoa, 2008). Third, and most notably, writing enjoyment functions as a modulator of intrinsic load perception (Plass and Kaplan, 2016). It does not alter the objective complexity but changes the learner’s subjective experience, making demanding tasks feel more manageable through competence-confidence loops and positive reappraisal (Pekrun, 2006). This finding supports and extends prior research of cognitive load and learning enjoyment in reading tasks (Cai et al., 2024) and with general learning tasks (Xu et al., 2020) by providing context-specific evidence within the complex domain of PBW.

### 5.2 The association between writing enjoyment and PBW achievement

The findings validate a partial mediation model in which writing enjoyment directly predicted writing achievement (*β* = 0.27, *p* < 0.01) and indirectly predicted it via cognitive load (*β* = 0.019, *p* = 0.006). This dual influence reinforces the central role of positive affect in academic achievement, particularly in cognitively demanding tasks such as PBW.

The direct positive association between writing enjoyment and PBW achievement aligns with a robust body of research linking writing enjoyment to improved writing achievement (Zumbrunn et al., 2019; Li et al., 2023). The interview data elucidate the behavioral mechanisms underlying this: writing enjoyment motivates increased learning investment (e.g., extensive academic reading), the adoption of writing strategies (e.g., collaborative peer discussion), heightened intrinsic motivation to pursue excellence, and sustained concentration during complex cognitive processes. These behaviors may have enhanced the quality of PBW output.

The indirect effect of writing enjoyment on PBW achievement, mediated by cognitive load, adds a critical cognitive dimension to the affective-achievement relationship in L2 writing. One possible explanation is that the lower cognitive load, negatively associated with writing enjoyment, may have left more working memory resources available for task-relevant processing (e.g., text construction, revision, and research design), which could contribute to better writing achievement. This supports and extends the findings of Tabari et al. (2024) in L2 writing and Xu et al. (2020) on growth mindset, confirming that the affective-cognitive interplay is a crucial lever for academic achievement in high-load environments.

### 5.3 Antecedents of writing enjoyment in PBW

The qualitative findings indicate that writing enjoyment in PBW was shaped by two closely related categories of antecedents: learner factors and situational factors.

Students’ writing enjoyment was strongly associated with perceived competence development. As they gradually mastered challenging tasks such as literature review, data analysis, and academic presentation, they reported stronger confidence, engagement, and satisfaction. This finding aligns with the IMCAL framework (Plass and Kaplan, 2016), which highlights the role of learners’ appraisals of competence and control in their emotional experiences during cognitively demanding learning activities.

Writing enjoyment was also supported by environmental conditions, especially topic-related interest and collaborative support. When students worked on topics they found meaningful and relevant, they were more likely to enjoy doing the task. This aligns with findings that personal interest in a topic can make the writing process more enjoyable and motivating (Alzubi and Nazim, 2024). Similarly, effective teamwork, clear task distribution, and encouragement from peers helped create a more positive learning experience. This finding agrees with previous literature in that students who often discussed with peers and received peer support, usually had higher writing enjoyment, or less anxiety (Zumbrunn et al., 2019).

The above findings extend prior research on emotion and cognitive load (Kalyuga, 2011; Plass and Kalyuga, 2019) by unpacking writing enjoyment antecedents in collaborative project writing tasks, offering practical references for PBW instructional design.

## 6 Implication and application

By demonstrating the partial mediation role of writing enjoyment in PBW achievement, the present study offers empirical support for Plass and Kaplan’s (2016) Integrated Model of Cognitive-Affective Learning (IMCAL) within the context of L2 academic writing. The findings suggest that positive affect, specifically writing enjoyment, extends beyond a peripheral role in complex PBW tasks, as reflected in its associations with cognitive load and writing achievement.

With regard to the teaching practice of PBW, this study offers pedagogical implications for fostering writing enjoyment. Interview data indicated that learner factors, such as mastery of specific skills and the achievement of completing challenging work, were the main antecedents of writing enjoyment. Accordingly, instructors may foster students’ writing enjoyment by enhancing their achievement experiences in PBW, for example, through teaching research skills (e.g., SPSS) or academic writing conventions. However, as Csikszentmihalyi (1990) noted, enjoyment is a positive state in which task challenges and the learner’s skills to meet them are well aligned. It is therefore important to establish language threshold requirements so that students have the capability to meet the challenges and enjoy the process of PBW exploration (Wang et al., 2023; Wang et al., 2024a).

Interview data also revealed several important situational factors contributing to writing enjoyment, including the freedom to choose writing topics, the chance to work in a supportive team, and the opportunity to engage in meaningful research. Based on these findings, we suggest that instructors adopt autonomy-supportive task design by allowing students to select PBW research topics aligned with their disciplines and personal interests. Topic autonomy, identified in this study as a core antecedent of writing enjoyment, can help sustain students’ engagement throughout the complex PBW process. Second, fostering collaborative learning environments and enhancing real-world task relevance may also be beneficial. For example, organizing small-group collaborative research and encouraging field research, data analysis, and the exploration of real-world research topics can enhance social interaction and task value. Third, given that acceptance of AI tools has been found to facilitate writing enjoyment or motivation (Sun et al., 2026a; Sun et al., 2026b) and foster a deep approach to learning (Sun et al., 2025), students are encouraged to make effective use of such tools in PBW tasks.

## 7 Conclusion

This study highlights the important role of writing enjoyment in PBW tasks: it positively predicts writing achievement and negatively predicts cognitive load, suggesting both direct and indirect associations with enhanced writing achievement. These findings extend the Integrated Model of Cognitive-Affective Learning (IMCAL) to L2 academic writing, support the integration of positive affect into cognitive load theory, and suggest that fostering writing enjoyment is a promising strategy for addressing the high cognitive demands of PBW.

Despite its theoretical and pedagogical contributions, this study has several limitations that point to directions for future research. First, the sample was limited to undergraduate students within a specific English proficiency range (high B1 to high C1), which constrains the generalizability of the findings. Future research should examine whether the relationships among writing enjoyment, cognitive load, and PBW achievement vary across learners with different English proficiency levels or at different educational stages (e.g., postgraduates or secondary school students). Second, writing achievement was measured through learners’ self-reports rather than objective, graded assessments by independent raters. Although this approach has been considered acceptable in previous research (Pike, 1996, 1999; Ross, 1998), self-reported achievement may be subject to systematic and random error, including the over- or under-estimation of one’s actual performance (Kuncel et al., 2005). Future studies should combine self-reported achievement with standardized writing rubrics and multiple-rater evaluations to enhance the validity of achievement measurement.

## Data Availability

The raw data supporting the conclusions of this article will be made available by the authors, without undue reservation.
